# Activation of LRP6 with HLY78 Attenuates Oxidative Stress and Neuronal Apoptosis via GSK3*β*/Sirt1/PGC-1*α* Pathway after ICH

**DOI:** 10.1155/2022/7542468

**Published:** 2022-04-04

**Authors:** Peng Jin, Dongqing Qi, Yuhui Cui, Cameron Lenahan, Shuixiang Deng, Xiaogen Tao

**Affiliations:** ^1^Department of Intensive Care Unit, The First Affiliated Hospital of USTC, Division of Life Sciences and Medicine, University of Science and Technology of China, Hefei, Anhui 230001, China; ^2^Department of Rehabilitation Medicine, The First Affiliated Hospital of USTC, Division of Life Sciences and Medicine, University of Science and Technology of China, Hefei, Anhui 230001, China; ^3^Department of Neurosurgery, Sixth People's Hospital Affiliated to Shanghai Jiao Tong University, Shanghai 200040, China; ^4^Burrell College of Osteopathic Medicine, Las Cruces, NM 88003, USA; ^5^Department of Intensive Care Unit, Huashan Hospital, Fudan University, Shanghai 200040, China

## Abstract

**Background:**

Oxidative stress and neuronal apoptosis have important roles in the pathogenesis after intracerebral hemorrhage (ICH). Previous studies have reported that low-density lipoprotein receptor-related protein 6 (LRP6) exerts neuroprotection in several neurological diseases. Herein, we investigate the role of LRP6 receptor activation with HLY78 to attenuate oxidative stress and neuronal apoptosis after ICH, as well as the underlying mechanism.

**Methods:**

A total of 199 CD1 mice were used. ICH was induced via injection of autologous blood into the right basal ganglia. HLY78 was administered via intranasal injection at 1 h after ICH. To explore the underlying mechanism, LRP6 siRNA and selisistat, a Sirt1 selective antagonist, were injected intracerebroventricularly at 48 h before ICH induction. Neurobehavioral tests, Western blot, and immunofluorescence staining were performed.

**Results:**

The expression of endogenous p-LRP6 was gradually increased and expressed on neurons after ICH. HLY78 significantly improved the short- and long-term neurobehavioral deficits after ICH, which was accompanied with decreased oxidative stress and neuronal apoptosis, as well as increased expression of p-GSK3*β*, Sirt1, and PGC-1*α*, as well as downregulation of Romo-1 and C-Caspase-3. LRP6 knockdown or Sirt1 inhibition abolished these effects of HLY78 after ICH.

**Conclusion:**

Our results suggest that administration of HLY78 attenuated oxidative stress, neuronal apoptosis, and neurobehavioral impairments through the LRP6/GSK3*β*/Sirt1/PGC-1*α* signaling pathway after ICH.

## 1. Introduction

Intracerebral hemorrhage (ICH) is one of the most dangerous subtypes of stroke and affects more than 1 million people annually [1, 2]. Despite extensive research into ICH pathology, a clinically approved neuroprotective treatment remains elusive [3]. There is growing evidence to suggest that overproduction of reactive oxygen species (ROS) serves as a key mechanism leading to brain injury after ICH [4]. Imbalance of ROS production and consumption leads to macromolecular damage, cellular signal transduction disorder, cell death, and tissue damage [5, 6]. Therefore, targeting oxidative stress and the subsequent neuronal apoptosis would be an effective way to improve the overall outcome of ICH.

Low-density lipoprotein receptor-related protein 6 (LRP6) is a Wnt coreceptor belonging to the low-density lipoprotein receptors (LDLRs) and is a transmembrane cell surface protein that induces the typical Wnt signaling pathway [7, 8]. LRP6 is involved in embryonic development, body fat, glucose homeostasis, and bone cell metabolism through induction of typical Wnt signaling pathways. Additionally, the role of LPR6 in cardiovascular system diseases has been extensively studied [9–12]. As research progressed, it was found that LRP6 activation could participate in neuroprotection in neurological disorders. Several studies have confirmed that LPR6 exerts neuroprotective effects through various mechanisms such as those that are anti-inflammatory, antioxidant, antiapoptotic, and protective of BBB integrity in focal cerebral ischemia and subarachnoid hemorrhage [13–15]. However, no studies have validated the neuroprotective role of LRP6 in brain injury after ICH.

HLY78 is a small molecule lycopene derivative that promotes LRP6 phosphorylation and Wnt signaling [16]. The antiapoptotic effect of HLY78 has also been demonstrated in tumors and in SAH [15, 17]. However, the role of HLY78 in ICH remains unexplored.

PGC-1*α* (peroxisome proliferator-activated receptor gamma coactivator 1-alpha) plays a key role in mitochondrial production and antioxidation [18]. Xu et al. demonstrated that PGC-1*α* exerts neuroprotective effects after SAH by entering the nucleus to promote antioxidative stress and antiapoptotic effects [19]. However, to date, few studies have explored the role of PGC-1*α* in ICH. Activation of PGC-1*α* requires Sirt1 (silent information regulator 1) acetylation [20]. Sirt1 reportedly improves mitochondrial function and attenuates brain injury by increasing PGC-1*α* levels [21]. Sirt1 interacts with GSK3*β*, and Koga et al. similarly found that Sirt1 upregulation can be mediated by GSK3*β* [22, 23] Under oxidative stress, LRP6 overexpression promoted the increased phosphorylation of GSK3*β* in cardiomyocytes [8].

In the current study, we hypothesized that LRP6 activation with HLY78 would attenuate mitochondria-mediated oxidative stress and neuronal apoptosis, thus improving neurological outcome after ICH. The potential mechanism may involve the GSK3*β*/Sirt1/PGC-1*α* signaling pathway.

## 2. Methods and Materials

All experimental protocols and procedures were approved by the Ethics Committee of Fudan University and in accordance with the National Institutes of Health guidelines.

### 2.1. Animals

Adult male CD1 mice weighing 25-35 g were purchased from Shanghai JieSiJie Laboratory Animal Co., Ltd. Mice were housed in a standard temperature and humidity-controlled room with a 12 h light/12 h dark cycle and ad libitum access to food and water.

### 2.2. ICH Model

The ICH model was induced via autologous blood injection as previously described [24]. Briefly, after deeply anesthetizing the mice with intraperitoneal injection of ketamine/xylazine (100/10 mg/kg), they were placed prone on a stereotactic head frame (Kopf Instruments, CA). Next, 30 *μ*l of autologous arterial blood collected from the left femoral artery was slowly injected into the right basal ganglia (coordinates: 1.5 mm lateral to the midline, 0.9 mm posterior to bregma, and 3.5 mm subdural) via a micro infusion pump (Harvard Instruments, MA). After injection, the needle remained in place for an additional 10 minutes to prevent extravasation of blood. Finally, the drill hole was sealed with bone wax, the skin incision was sutured, and the mice were allowed to recover. The body temperature of the mice was maintained throughout the procedure using an electronic thermostat-controlled heating blanket (37 ± 0.5°C). The sham mice underwent the same surgical procedure, except for blood injection.

### 2.3. Drug Administration

#### 2.3.1. Intranasal Administration

HLY78 or DMSO was administered via intranasal (i.n) injection while under isoflurane anesthesia at 1 h after ICH as previously described [25]. Specifically, DMSO (vehicle) or HLY78 (0.6 mg/kg, 1.2 mg/kg, and 2.4 mg/kg) was administered as nose drops (5 *μ*L/drop) over a period of 30 min, alternating drops every 5 min between the left and right nares. The total volume delivered in each mouse was 30 *μ*L.

#### 2.3.2. Intracerebroventricular Injection

LRP6 siRNA (500 pmol/5 *μ*l; OriGene Technologies, MD, USA), scrambled siRNA (5 *μ*l), or selisistat was injected via intracerebroventricular injection (i.c.v.) at 48 h prior to surgery as previously described [26]. After being deeply anesthetized, the mice were placed in a stereotaxic apparatus. The needle was inserted into the left lateral ventricle (coordinates relative to bregma: 0.3 mm posterior, 1.0 mm lateral, and 2.3 mm deep) through a burr hole on the skull. The drugs were administrated at a rate of 0.5 *μ*l/min using an infusion pump. After injection, the needle remained in the same position for another 10 minutes to prevent leakage. After remaining in place for 10 minutes, it was slowly withdrawn over a period of 5 minutes.

### 2.4. Experiment Design

**Figure 1:**
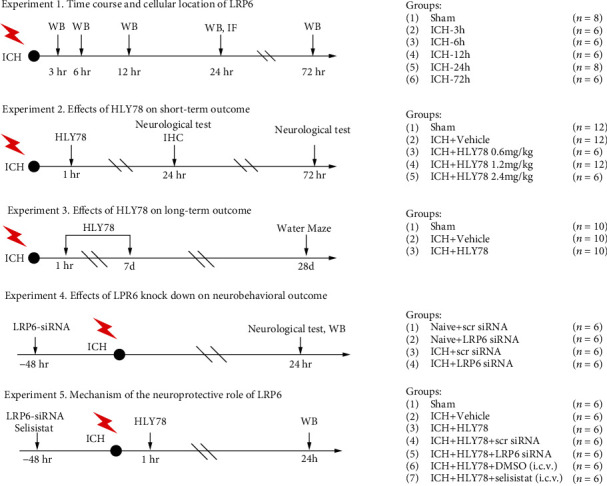
Experimental design and animal groups. ICH: intracerebral hemorrhage; WB: Western blot; IF: immunofluorescence; DMSO: dimethyl sulfoxide; i.c.v.: intracerebroventricularly.

A total of five separate experiments were performed as depicted in Figure 1.

#### 2.4.1. Experiment 1. Time-Course and Cellular Localization

To determine the endogenous protein levels and cellular localization of p-LRP6, 40 mice were randomly divided into 6 groups: sham (*n* = 8), ICH-3 h (*n* = 6), ICH-6 h (*n* = 6), ICH-12 h (*n* = 6), ICH-24 h (*n* = 8), and ICH-72 h (*n* = 6). The p-LRP6 protein levels were detected by Western blot in the right cerebral hemisphere, and the cellular localization of p-LRP6 was assessed via immunofluorescence staining.

#### 2.4.2. Experiment 2. Effect of HLY78 Treatment on Short-Term Neurobehavioral Outcome after ICH

To determine the effect of HLY78 on neurological outcome and neuronal damage in ICH mice, 30 mice were assigned to five groups: sham, ICH+vehicle, ICH+HLY78 (0.6 mg/kg), ICH+HLY78 (1.2 mg/kg), and ICH+HLY78 (2.4 mg/kg). Neurological testing (modified Garcia test, corner turn test, and forelimb placement test) was assessed at 24 h after ICH. The optimal drug dose was selected based on the results of the 24-hour behavioral experiment, and then, 18 mice were randomly divided into sham, ICH+vehicle, and ICH+HLY78 (best dose). Brain tissues were removed for DHE staining, FJC staining, TUNEL staining, and Western blot at 24 hours after surgery to evaluate the effects of HLY78 on neuronal damage and oxidative stress. An additional 18 mice were randomly divided into three groups: sham, ICH+vehicle, and ICH+HLY78 (best dose) to further assess the ameliorative effect of HLY78 on neurological deficits in ICH mice at 72 hours.

#### 2.4.3. Experiment 3. Effect of HLY78 Treatment on Long-Term Neurobehavioral Outcome after ICH

To investigate the effects of HLY78 on long-term neurobehavioral function after ICH, 30 mice were randomly assigned into three groups: sham, ICH+vehicle, and ICH+HLY78 (best dose) (*n* = 10/group). The foot fault and rotarod tests were performed on weeks 1-3, and the Morris water maze was conducted on days 23–28 after ICH.

#### 2.4.4. Experiment 4. Effect of LRP6 on Neurological Deficits after ICH

LRP6 siRNA was used to explore the role of LRP6 on neurological deficits after ICH. Twenty-four mice were randomly assigned to four groups: naïve+scr siRNA, naïve+LRP6 siRNA, ICH+scr siRNA, and ICH+LRP6 siRNA (*n* = 6/group). Western blots and neurobehavioral tests were performed 24 hours after ICH.

#### 2.4.5. Experiment 5. Potential Neuroprotective Molecular Mechanism of HLY78 after ICH

To explore the mechanism of the neuroprotective role of HLY78, 42 mice were randomly divided into seven groups: sham, ICH+vehicle, ICH+HLY78, ICH+HLY78+scr siRNA, ICH+HLY78+LRP6 siRNA, ICH+HLY78+DMSO, and ICH+HLY78+selisistat (*n* = 6/group). The expression of GSK3*β*, p-GSK3*β*, Sirt1, PGC-1*α*, Romo-1, and C-Caspase-3 was assessed via Western blot 24 h after ICH.

### 2.5. Neurobehavioral Test

Neurobehavioral tests were performed by an investigator that was blinded from the experimental group's information.

#### 2.5.1. Short-Term Neurobehavioral Test

Three behavioral tests were applied at 24 and 72 h after ICH: modified Garcia test, corner turn test, and forelimb placement test, as previously described [27].

The modified Garcia test consists of seven separate tests including spontaneous activity, symmetry of limb movement, forelimb extension, climbing, proprioception, response to a vibrating screen, and lateral rotation. Each of these has a possible score of 3, with a maximum score of 21 representing optimal neurobehavioral function.

The forelimb placement test was performed by holding the mouse's trunk and then bringing its left vibrissae into contact with the edge of the table. The results were recorded as the percentage of left forelimb placements on the platform out of the total number of vibrissae strokes.

The corner test was performed in a 30° corner formed by two transparent plates. The mouse was placed on the platform and guided to turn in the corner. The result was recorded as a percentage of the behavior of the left turn.

#### 2.5.2. Long-Term Neurobehavioral Test

Foot fault tests were used to assess sensory function and were conducted on weeks 1-3 after ICH, as previously described [28]. The mice were allowed to move freely for 2 minutes on a wire mesh covered with 2 cm × 2 cm squares. The number of missed steps in which the mouse's left forelimb slipped through one of the square holes was recorded.

The rotarod test is commonly used to assess motor coordination in animals [29]. The mice were placed on the rotating cylinder, the starting speed was set at 5 RPM, and the time of the mice falling was recorded for 5 consecutive times. The average time of the 5 times was finally taken.

Morris water maze experiments were performed on days 23 to 28 after ICH to assess the learning and memory functions of the mice as previously described [30]. During the first 5 days of the experiment, the mice were randomly placed in the pool from different points each time until they swam to the platform selectively placed in a quadrant of the pool and remained there. The distance they swam and the time it took to find the platform were recorded. On the 6th day of the experiment, the platform was removed and the mice were placed into the water to swim for 60 s. The movement trajectory of the mice and the time spent in the platform quadrant were recorded.

### 2.6. Immunofluorescence Staining

#### 2.6.1. Histology

After being deeply anesthetized, the mice were transcardially perfused with PBS, followed by 10% formalin. Then, the brains were removed and placed in 10% formalin for 1 day, followed by placement in 30% sucrose solution for 3 days until the brains were frozen in OCT and sliced into 10 *μ*m coronal sections using a cryostat. These sections were stored at -80°C for subsequent experiments.

#### 2.6.2. Double Immunofluorescence Staining

Double immunofluorescence staining was performed as previously described [31]. After being washed 3 times with 0.01 M PBS for 10 minutes each, the slides were then incubated in 0.3% Triton X-100 for 10 minutes, followed by incubation in 5% donkey serum at room temperature for 2 hours. The brain sections were then incubated overnight at 4°C with the following primary antibodies: anti-p-LRP6 (1 : 50, MyBioSource Inc., USA) and anti-NeuN (1 : 100, Abcam, USA). The sections were then washed with PBS and incubated with the corresponding secondary antibody (1 : 100, Jackson Immuno Research, USA) for 1 hour at room temperature. Lastly, they were visualized and photographed with a fluorescence microscope (Leica Microsystems, Germany).

#### 2.6.3. DHE Staining

Dihydroethidium (DHE) staining was conducted to evaluate ROS production as previously described [32]. The brain sections were incubated with freshly prepared dihydroethidium (2 *μ*mol/L) (Thermo Fisher Scientific, USA) for 30 min at 37°C in the dark. The images were captured using a fluorescence microscope, and the fluorescence intensity was quantified with the ImageJ software.

#### 2.6.4. TUNEL Staining

Terminal deoxynucleotidyl transferase dUTP nick end labeling (TUNEL) staining was conducted using the Apoptosis Detection Kit (Roche, USA) according to the manufacturer's instructions for quantification of the apoptosis of neurons surrounding the hematoma 24 hours after ICH [33]. The TUNEL-positive neurons were counted manually at ×200 magnification in the perihematomal region. Data were expressed as the ratio of TUNEL-positive neurons (%).

#### 2.6.5. FJC Staining

Degenerated neurons were stained using the Fluoro-Jade C (FJC) Ready-to-Dilute Staining Kit (Biosensis, USA) according to the manufacturer's instructions. FJC-positive neurons were counted in six sections of each brain using the ImageJ software to assess the extent of neuronal damage. Data were expressed as cells/mm^2^ as the average number of FJC-positive neurons in each region.

### 2.7. Western Blot

Western blot was performed as previously described [34]. After being deeply anesthetized and transcardially perfused with frozen PBS, the right hemisphere of the mouse brain was collected and stored at -80°C until use. The brain tissue was homogenized in RIPA lysis buffer (Santa Cruz Biotechnology Inc., USA) and then centrifuged at 12,000 RPM at 4°C for 30 min, and the supernatant was normalized to a solution with equal protein concentration. The protein solution was mixed with loading buffer reagent and then denatured (95°C, 10 min). Protein samples with equal volumes were loaded on an SDS-PAGE gel, and then, the electrophoresed protein bands were transferred to a nitrocellulose membrane. The membrane was incubated with the following primary antibodies overnight at 4°C: anti-p-LRP6 (1: 250, MyBioSource, USA), anti-LRP6 (1 : 1000, MyBioSource, USA), p-GSK3*β* (1 : 500, Cell Signaling Technology, USA), GSK3*β* (1 : 1000, Cell Signaling Technology, USA), Sirt1 (1 : 2000, Abcam, USA), PGC-1*α* (1 : 1000, Abcam, USA), cleaved caspase-3 (1 : 250, Abcam, USA), anti-Romo-1 (1 : 200, AVIVA Systems Biology, USA), and anti-*β*-actin (1 : 5000, Santa Cruz Biotechnology Inc., USA). Then, the appropriate secondary antibody (Santa Cruz Biotechnology, USA) was applied to the membranes and incubated for 1 h at room temperature. Immunoblots were then visualized with ECL Plus chemiluminescence reagent kit (Amersham Bioscience, USA) and quantified using the ImageJ software.

### 2.8. Statistical Analysis

All data were expressed as the mean and standard deviation (mean ± SD) and analyzed with GraphPad Prism (GraphPad Software, USA). Multiple comparisons were analyzed using one-way ANOVA and post hoc Bonferroni test. Two-way ANOVA, followed by Tukey post hoc test, was used to compare the changes according to the different levels of multiple categorical variables. Data were screened for normality using the Shapiro-Wilk test. Since data were not normally distributed, the between-group differences were analyzed with nonparametric test. *p* < 0.05 was considered to be statistically significant.

## 3. Results

### 3.1. Animal Use and Mortality

A total of 199 adult CD1 mice were used in our experiment. Of which, 168 underwent blood injection and 30 underwent sham surgery. No mice died in the sham group, and 11 died in the ICH group, with an overall mortality rate of 5.53% (11/199).

### 3.2. Time Course and Cellular Location of Endogenous p-LRP6 in Ipsilateral Hemisphere after ICH

**Figure 2:**
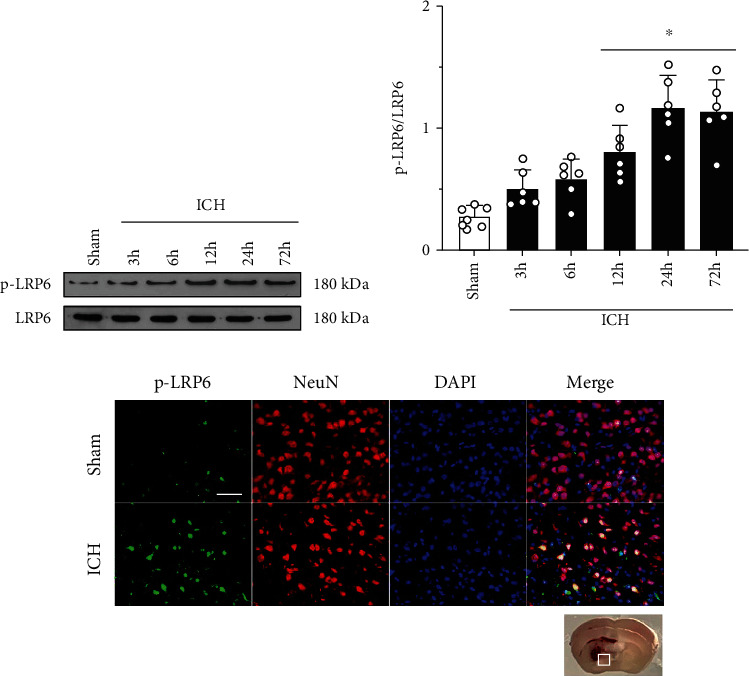
Time course and cellular location of p-LRP6 after ICH. (a) Representative Western blot bands of p-LRP6 in the ipsilateral hemisphere after ICH. (b) Quantitative analyses of p-LRP6 in the ipsilateral hemisphere after ICH. Data was represented as mean ± SD. ^∗^*p* < 0.05 vs. sham group; one-way ANOVA and Tukey test, *n* = 6/group. (c) Representative microphotographs of p-LRP6 (green) and neurons (NeuN, red) in the perihematomal area 24 h after ICH. Scale bar = 50 *μ*m, *n* = 2/group.

The endogenous expression of p-LRP6 was assessed by Western blot at 0 (sham), 3, 6, 12, 24, and 72 h in the ipsilateral cerebral hemispheres after ICH. Compared to the sham group, endogenous p-LRP6 protein levels began increasing 3 hours after ICH, peaked at 24 hours, and persisted until 72 hours after ICH (Figures 2(a) and 2(b)). To confirm the cellular location of p-LRP6, double immunofluorescence staining was performed at 24 hours after ICH, as the protein expression peaked at this time point. The results showed that p-LRP6 was abundantly expressed on neurons, and the number of p-LRP6-positive neurons increased significantly after ICH (Figure 2(c)).

### 3.3. HLY78 Improved Short-Term Neurological Deficits at 24 h after ICH

**Figure 3:**
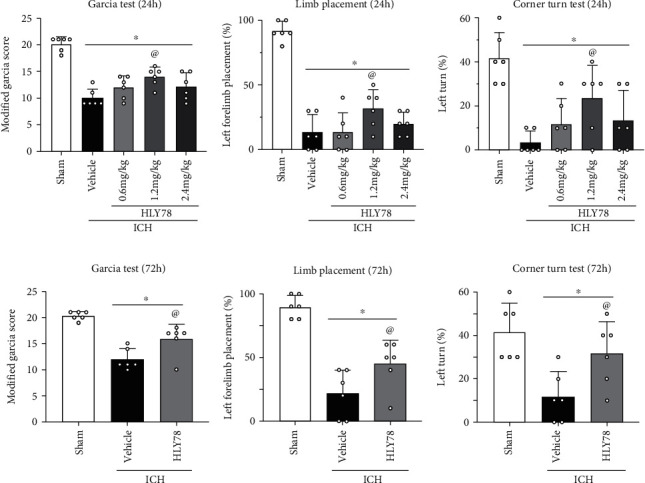
The effects of HLY78 on short-term neurological functions at 24 and 72 h after ICH. (a–c) Modified Garcia test, forelimb placement test, and corner turn test at 24 h after ICH. (d–f) Modified Garcia test, forelimb placement test, and corner turn test at 72 h after ICH. Data was represented as mean ± SD. ^∗^*p* < 0.05 vs. sham; ^@^*p* < 0.05 vs. ICH+vehicle. One-way ANOVA and Tukey test, *n* = 6/group.

To assess the effects of HLY78 on neurological deficits and to determine the optimal dose, three different doses of HLY78 were administered intranasally at 1 hour after ICH. Neurological outcomes were assessed by modified Garcia test, corner turn test, and forelimb placement test at 24 and 72 hours after ICH. The results showed that when compared to the sham group, mice in the ICH group exhibited significant neurological deficits, but administration of HLY78 (1.2 mg/kg) significantly improved neurological outcomes at 24 hours after ICH compared to the ICH+vehicle group (*p* < 0.05 Figures 3(a)–3(c)). To further validate the therapeutic effect of HLY78 (1.2 mg/kg), neurobehavioral testing was also performed at 72 hours post-ICH. Consistently, the ICH+HLY78 (1.2 mg/kg) group demonstrated improved neurological function at 72 hours post-ICH when compared to the ICH+vehicle group (*p* < 0.05 Figures 3(d)–3(f)). Based on these results, the moderate dose of HLY78 (1.2 mg/kg) was selected as the optimal dose in the subsequent study.

### 3.4. HLY78 Reduces Oxidative Stress Injury in the Perihematomal Area 24 Hours after ICH

**Figure 4:**
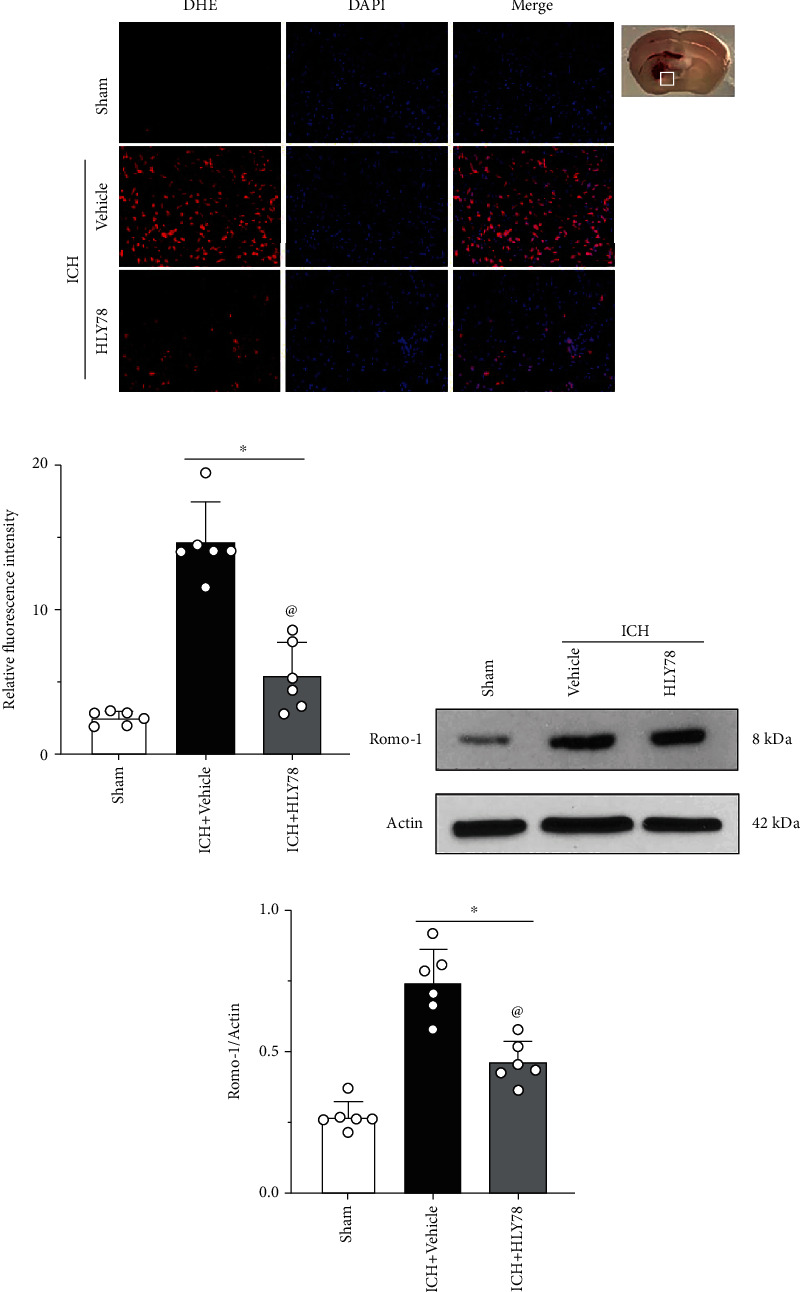
Effects of HLY78 on oxidative stress level of brain at 24 h after ICH. (a) Representative microphotographs of DHE staining in the perihematomal area 24 h after ICH. Scale bar = 100 *μ*m. (b) Quantitative analysis of DHE fluorescence intensity. (c) Representative Western blot bands of Romo-1 in the ipsilateral hemisphere after ICH. (d) Quantitative analyses of Romo-1 in the ipsilateral hemisphere after ICH. ^∗^*p* < 0.05 vs. sham group; ^@^*p* < 0.05 vs. ICH+vehicle group. One-way ANOVA and Tukey test, *n* = 6/group.

The level of oxidative stress in the perihematomal area after ICH was measured by DHE staining, and the expression of Romo-1 was detected by Western blot. The intensity of DHE was significantly increased in the ICH+vehicle group at 24 hours after ICH compared with the sham group. Intranasal administration of HLY78 significantly reduced the intensity of DHE compared to the control group (*p* < 0.05 Figures 4(a) and 4(b)). Romo-1 is a marker of oxidative stress. Our data showed that Romo-1 was significantly increased in the vehicle group compared to the sham group at 24 hours after ICH, and treatment with HLY78 significantly decreased the level of Romo-1 (*p* < 0.05 Figures 4(c) and 4(d)).

### 3.5. HLY78 Reduced Neuronal Apoptosis and Neuronal Degeneration at 24 h after ICH

**Figure 5:**
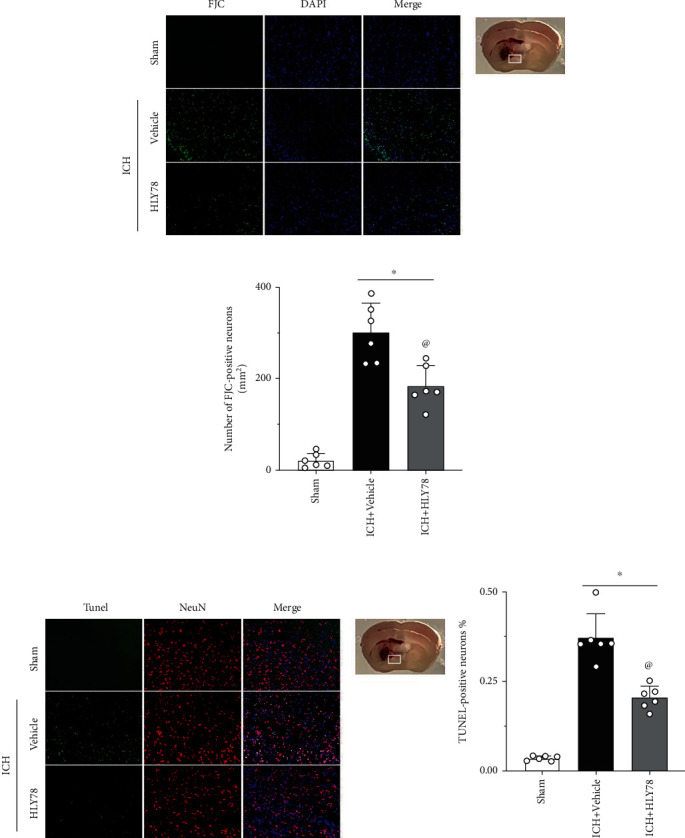
Effects of HLY78 on neuronal damage at 24 h after ICH. (a) Representative micrographs of FJC staining in the perihematomal area, and (b) quantitative analysis of FJC-positive neurons 24 h after ICH. (c) Representative micrographs of TUNEL staining in the perihematomal area, and (d) quantitative analysis of TUNEL-positive neurons. 24 h after ICH. Scale bar = 100 *μ*m. ^∗^*p* < 0.05 vs. sham group; ^@^*p* < 0.05 vs. ICH+vehicle group. One-way ANOVA and Tukey test, *n* = 6/group.

**Figure 6:**
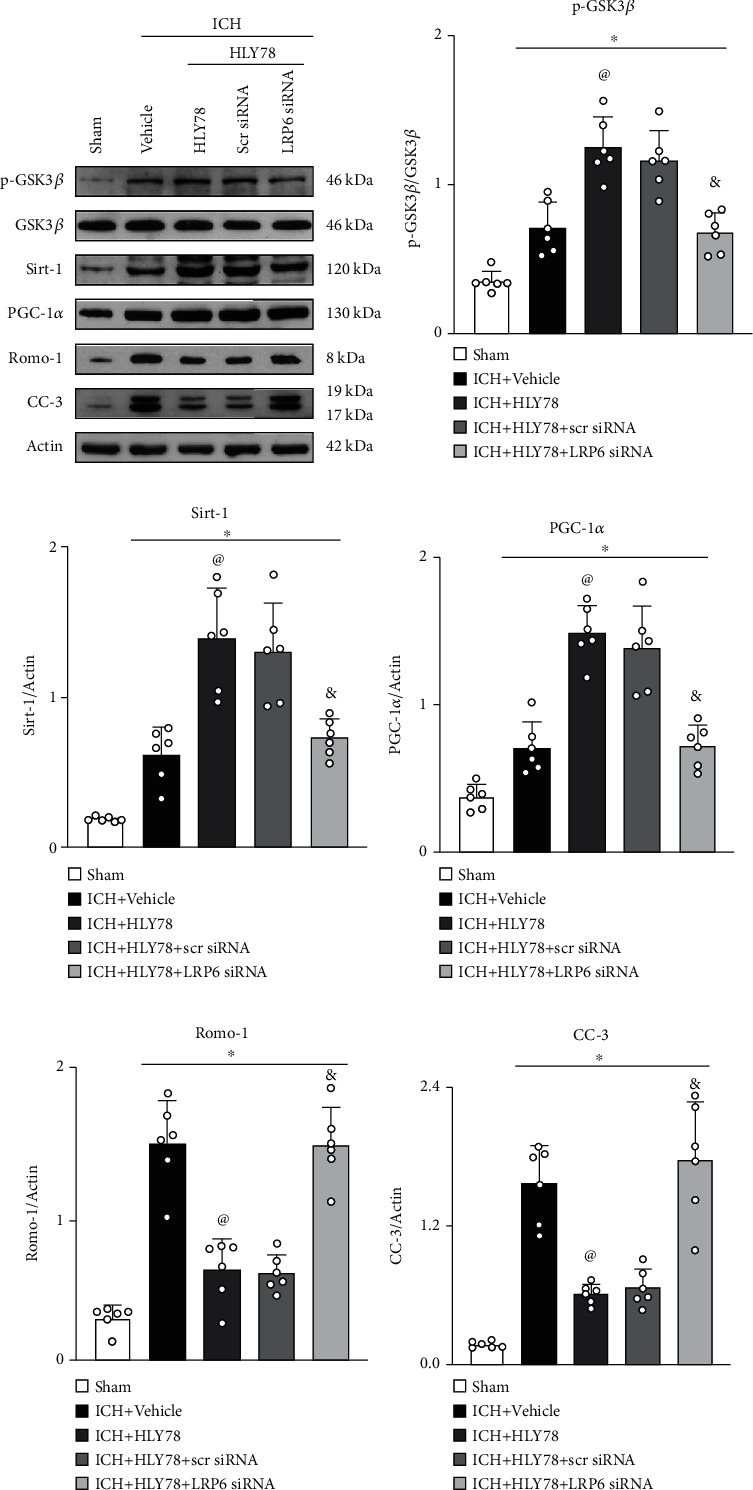
LRP6 siRNA blunt the protective effects of HLY78 at 24 h after ICH. (a) Representative Western blot images. (b–f) Quantitative analyses of p-GSK3*β*, Sirt1, PGC-1*α*, Romo-1, and C-Caspase-3. Data are represented as mean ± SD. ^∗^*p* < 0.05 vs. sham, ^@^*p* < 0.05 vs. ICH+vehicle, and ^&^*p* < 0.05 vs. ICH+HLY78+scr siRNA. One-way ANOVA and Tukey's post hoc test, *n* = 6/group.

Since ICH could cause neuronal apoptosis and degeneration, TUNEL staining and FJC staining were used to determine whether HLY78 reduced damage 24 hours after ICH. Compared with the sham group, TUNEL-positive neurons and FJC-positive neurons in the perihematomal area were significantly increased in the ICH+vehicle group at 24 hours after ICH, but HLY78 treatment reduced the number of TUNEL-positive and FJC-positive neurons (*p* < 0.05 Figures 5(a)–5(d)). Western blot data showed that C-Caspase-3 was significantly increased after ICH, and that HLY78 treatment could reduce the expression of C-Caspase-3 (Figures 6(a) and 6(f)). These results suggest that HLY78 treatment can significantly reduce neuronal damage in ICH.

### 3.6. HLY78 Treatment Improved Long-Term Neurological Outcomes after ICH

**Figure 7:**
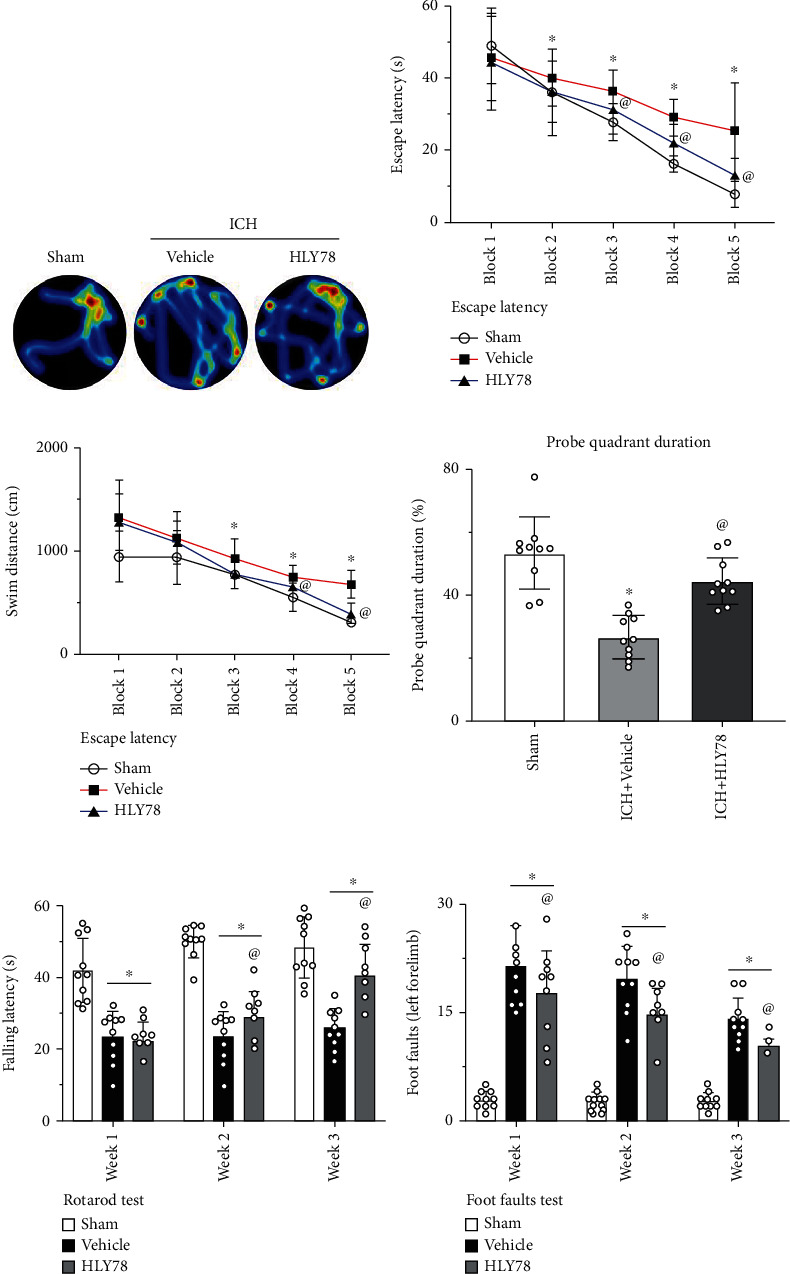
Effect of HLY78 on long-term neurological function after ICH. (a) Representative heatmaps of the probe trial. (d) Quantification of the probe quadrant duration in the probe trial. (b) Escape latency and (c) swimming distance of Morris water maze. (e) Rotarod test and (f) foot fault test at day 7, 14, and 21 post-ICH. Data was represented as mean ± SD. ^∗^*p* < 0.05 vs. sham; ^@^*p* < 0.05 vs. ICH+vehicle group. Two-way repeated-measures ANOVA and Tukey's post hoc test, *n* = 10/group.

To evaluate the effect of HLY778 on long-term functions after ICH, Morris water maze tests were performed from days 23-28 after surgery. Compared with the sham group, mice in the ICH+vehicle group swam longer distances and spent significantly longer time searching for the platform on days 3, 4, and 5 when compared to the sham group. In contrast, mice in the HLY78-treated group demonstrated shorter swimming distances and time spent searching for the platform (*p* < 0.05 Figures 7(b) and 7(c)). In the probe quadrant trial, the ICH+vehicle group spent less time in the target quadrant when the platform was removed compared to the sham group, while the time spent in the target quadrant was significantly increased in the ICH+HLY78 group compared to the ICH+vehicle group (*p* < 0.05 Figures 7(a) and 7(d)). These results showed that HLY78 significantly improved memory and learning dysfunction after ICH.

Regarding the rotarod test, the mice in the ICH+vehicle group had significantly shorter falling latency than the sham group. Consistently, the ICH+vehicle group had more foot faults of the left forelimb when compared with sham groups in foot fault tests after ICH. However, HLY78 treatment significantly improved the behavioral performance in foot fault test and rotarod test in ICH mice (*p* < 0.05, Figures 7(b) and 7(c)).

### 3.7. Endogenous Brain LRP6 Knockdown Exacerbated Neurological Deficits at 24 h after ICH

**Figure 8:**
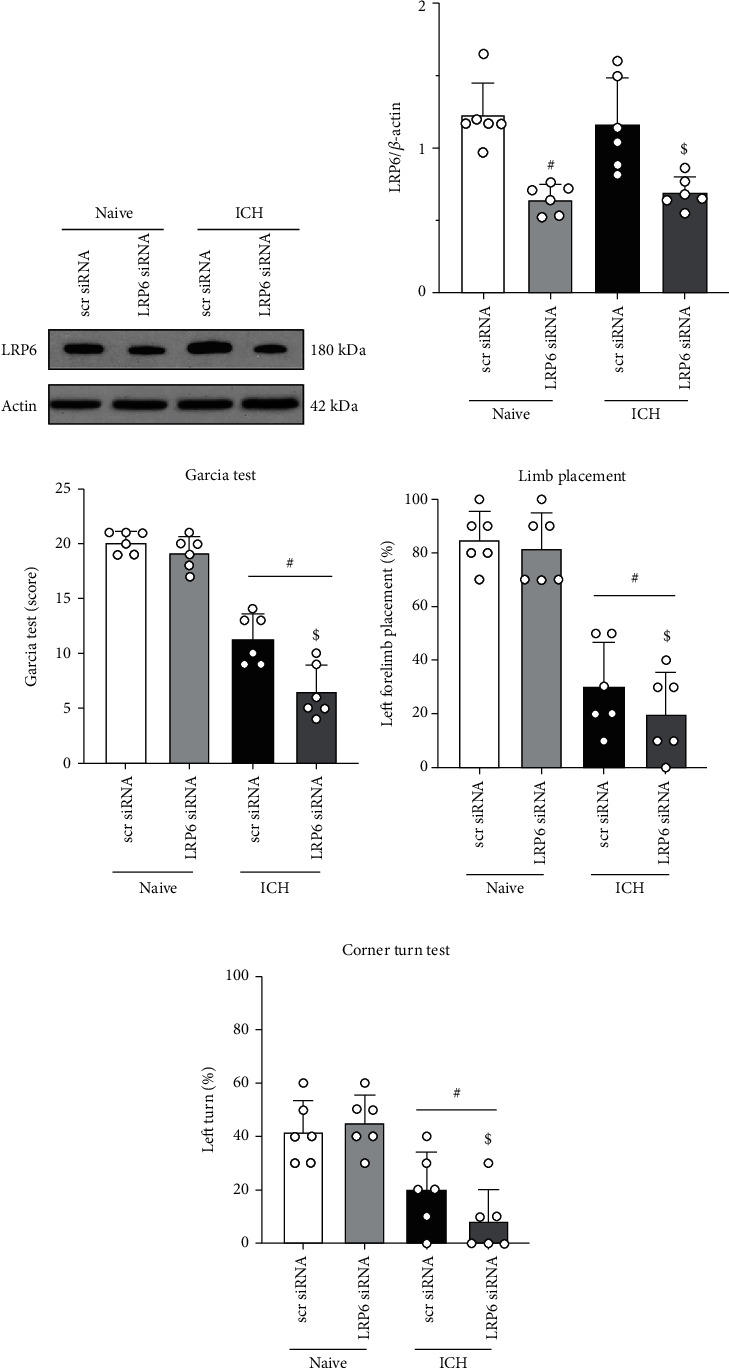
Effects of LRP6 knock down with siRNA on the level of LRP6 and neurological function at 24 h after ICH. (a) Representative Western blot bands of LRP6 after administration of LRP6 siRNA, and (b) quantitative analyses of LRP6. (c–e) Modified Garcia test, forelimb placement test, and corner turn test at 24 h after surgery. Data was represented as mean ± SD. ^#^*p* < 0.05 vs. naïve+scr siRNA and ^$^*p* < 0.05 vs. ICH+scr siRNA. One-way ANOVA and Tukey test, *n* = 6/group.

To assess the role of endogenous LRP6 after ICH, LRP6 siRNA was administered 48 h before ICH induction. Western blot results showed that the protein level of LRP6 was significantly decreased in LRP6 siRNA-treated naïve mice or ICH mice when compared with the scr siRNA group (*p* < 0.05, Figures 8(a) and 8(b)), indicating the LRP6 knockdown efficiency of siRNA. In naïve mice, LRP6 knockdown did not adversely affect the neurological function of the mice, whereas in ICH mice, LRP6 knockdown exacerbated neurobehavioral deficits (*p* < 0.05, Figures 8(c) and 8(d)). Taken together, these results suggest its role as an endogenous protective response to stress and deleterious stimuli in the acute phase after ICH.

### 3.8. HLY78 Attenuates Oxidative Stress and Neuronal Apoptosis via LPR6/GSK3*β*/Sirt1/PGC-1*α* Signaling Pathway at 24 h after ICH

Western blot data showed that the expression of p-GSK3*β*, Sirt1, PGC-1*α*, Romo-1, and C-Caspase-3 was significantly increased 24 hours after ICH when compared with the sham group. HLY78 treatment further increased the expression of p-GSK3*β*, Sirt1, and PGC-1*α*, but the expression of C-Caspase-3 and Romo-1 was decreased in the ICH+HLY78 group when compared with the ICH+vehicle group. LRP6 siRNA was given 48 hours prior to ICH induction to knock down the gene of LRP6, which was done to assess the involvement of LRP6 in the antiapoptotic and antioxidant pathways of HLY78 after ICH. Our data showed that pretreatment with LRP6 siRNA significantly reduced the downstream molecules p-GSK3*β*, Sirt1, and PGC-1*α* when compared to the ICH+HLY78+scr siRNA group. However, compared to the control group, the ICH+HLY78+LRP6 siRNA group observed a significant overexpression of C-Caspase-3 and Romo-1 expression (*p* < 0.05, Figures 6(a)–6(f)). These results suggest that HLY78 exerts antiapoptotic and antioxidant neuroprotective effect mediated through LRP6.

**Figure 9:**
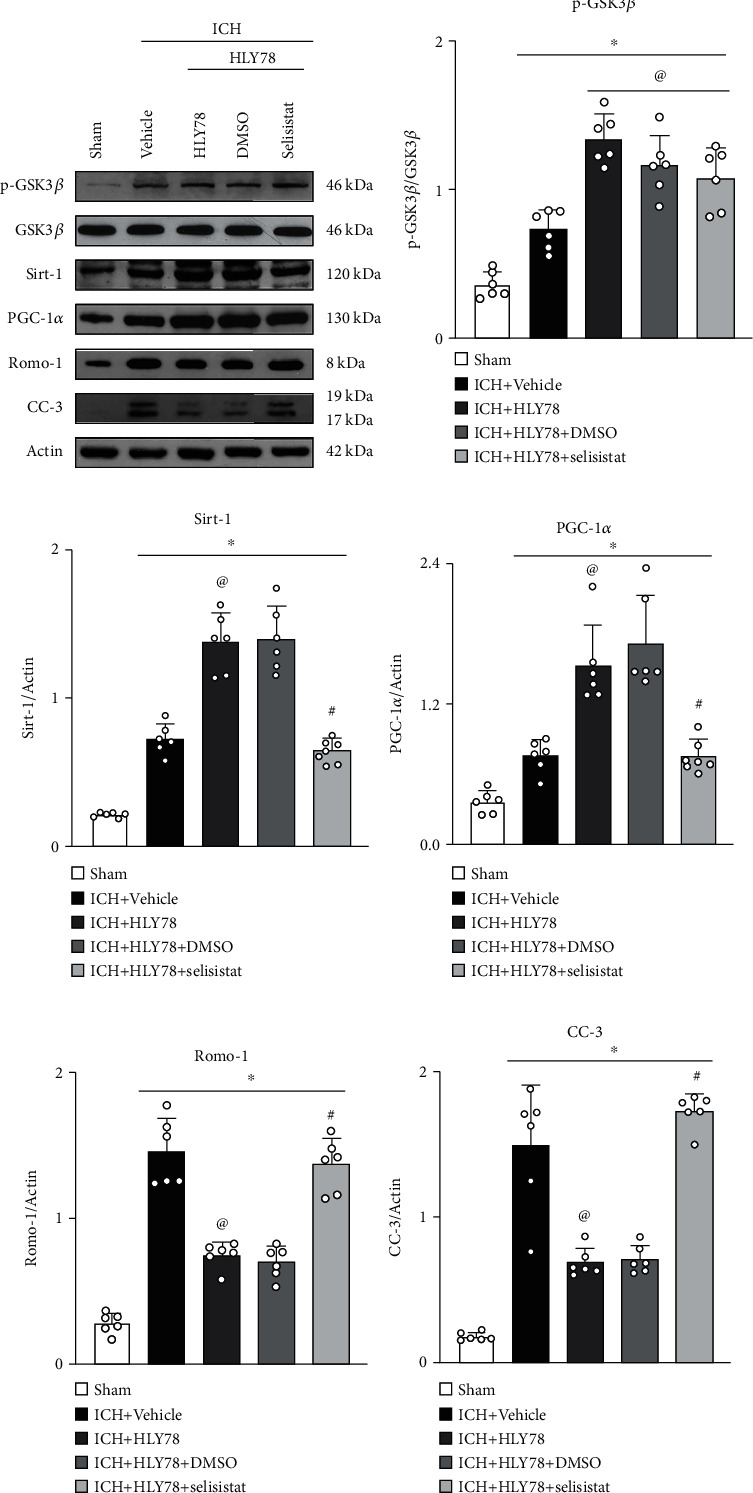
Sirt1 inhibitor, selisistat, reversed the effects of HLY78 at 24 h after ICH. (a) Representative Western blot images. (b–f) Quantitative analyses of p-GSK3*β*, Sirt1, PGC-1*α*, Romo-1, and C-Caspase-3. Data are represented as mean ± SD. ^∗^*p* < 0.05 vs. sham, ^@^*p* < 0.05 vs. ICH+vehicle and ^#^*p* < 0.05 vs. ICH+HLY78+DMSO. One-way ANOVA and Tukey's post hoc test, *n* = 6/group.

To verify the role of Sirt1 in LRP6-mediated neuroprotective effects, selisistat, a specific inhibitor of Sirt1, was administered. Western blot analysis showed that selisistat did not affect the protein expression level of GSK3*β*. However, the levels of Sirt1 and PGC-1*α* were significantly decreased, whereas the levels of C-Caspase-3 and Romo-1 were increased in the ICH+HLY78+selisistat group when compared with the ICH+HLY78+DMSO group (*p* < 0.05, Figures 9(a)–9(f)). Thus, Sirt1 acts as a downstream factor of LRP6, and its inhibitory effect abolishes the antiapoptotic and antioxidative stress effects of HLY78. These results suggest that LRP6 activation by HLY78 attenuated oxidative stress, neuronal apoptosis, and neurological deficits after ICH in mice, partially through the GSK3*β*/Sirt1/PGC-1*α* signaling pathway.

## 4. Discussion

Intracerebral hemorrhage (ICH) is a stroke subtype associated with high rates of mortality and disability. Oxidative stress and neuronal apoptosis play important roles in the pathogenesis of early brain injury (EBI) after ICH [5, 35]. Therefore, inhibition of oxidative stress and neuronal apoptosis are potential therapeutic targets for ICH-induced secondary brain injury (SBI), which would provide a promising strategy for clinical treatment. In the present study, we focused on the effects of HLY78 treatment in reducing OS-induced neuronal degeneration and apoptosis after ICH. The major novel findings of this study are as follows: (1) endogenous expression of p-LRP6 in brain was abundantly expressed on neurons, which increased in a time-dependent manner and peaked at 24 h after ICH; (2) intranasal administration of HLY78 remarkably reduced oxidative stress and neuronal apoptosis 24 h after ICH and improved short- and long-term neurological deficits; (3) HLY78 treatment was associated with upregulation of p-GSK3*β*, Sirt1, and PGC-1*α* and downregulation of C-Caspase-3 and Romo-1 at 24 h after ICH. (4) Silence of LRP6 or inhibition of Sirt1 partially reversed the effects of HLY78 on the expression of p-GSK3*β*, Sirt1, PGC-1*α*, C-Caspase-3, and Romo-1. These results suggest that HLY78 is neuroprotective against OS-induced neuronal damage after ICH. This protection is exerted at least partially through the LRP6/GSK3*β*/Sirt1/PGC-1*α* signaling pathway.

LRP6 belongs to the low-density lipoprotein (LDL) receptor-related family and is a transmembrane cell surface protein. After Wnt binds to its receptor, Frizzled, LRP6 forms a complex with Wnt-Frizzled to exert biological effects and promote the transcription of target genes [7]. Its molecular mechanism involves a variety of biological processes, including cancer development, immune response, or cellular metabolism [36, 37]. A study by Wang et al. indicated that LRP6 could improve myocardial ischemia after refocusing injury through antioxidant and antiapoptotic effects [8, 38]. In recent years, the role of LRP6 in neurological diseases has also been gradually investigated. The role of LRP6 is crucial in embryonic development and synapse formation, and loss of LRP6 leads to severe neurological deficits, including reduced production of granule cells in the hippocampus, defects in thalamic development, and impaired neocortical neuronal proliferation [39, 40]. Liu et al. found that LRP6 mRNA and protein levels are significantly downregulated compared to age-matched controls in human AD brains [41]. Likewise, LRP6 haploinsufficiency increases proinflammatory markers, mitochondrial dysfunction, and stroke volume [13]. Harriott et al. found that the LRP6 variant was associated with the risk of ischemic stroke [42]. However, to date, there have not been any studies to confirm the changes in LRP6 expression and its neuroprotective effects after ICH. Our study found that p-LRP6 protein expression levels were significantly upregulated and abundantly expressed on neurons after ICH, which is consistent with the previous findings [14, 15]. Thus, the temporal elevation of p-LRP6 protein expression may be an endogenous neuroprotective mechanism after ICH. However, this upregulation was not sufficient to counteract the overall brain injury in ICH mice. To further confirm this hypothesis, we used siRNA to knock down LRP6 protein expression before surgery, and the Western blot results showed that LRP6 siRNA administration significantly downregulated the LRP6 protein expression. Further neurobehavioral experiments confirmed that in naïve mice, knocking down LRP6 did not have an effect on neurological function, whereas in ICH mice, knocking down LRP6 clearly exacerbated the neurological deficits found in the mice. These results likewise suggest that LRP6 plays a neuroprotective role in ICH.

HLY78 is a novel lycorine derivative and has previously been shown to promote LRP6 phosphorylation and Wnt/*β*-catenin signaling by enhancing Axin-LRP6 association [43]. In the SAH model, intranasal administration of HLY78 also upregulated the expression of p-LRP6 protein, which in turn functioned as an antiapoptotic agent and protected the integrity of the BBB [14, 15]. However, the role of HLY78 in ICH has not yet been explored. Intranasal administration of drugs is a noninvasive method that can bypass the BBB for delivering drugs that target central nervous system disorders [44, 45]. Therefore, we administered HLY78 intranasally at 1 hour after ICH in this study. The results showed that HLY78 treatment significantly improved short- and long-term outcome, improved deficits in learning and memory found in ICH mice, and attenuated oxidative stress levels and neuronal damage in the perihematomal area. These results suggest that HLY78 may be an effective drug for treating ICH.

Then, we studied the underlying mechanisms behind LRP6-mediated antioxidative stress and antiapoptotic effects. Under oxidative stress, LRP6 overexpression promoted the increased phosphorylation of GSK3*β* in cardiomyocytes [46]. Luo et al. demonstrated that LRP6 has a neuroprotective role in ischemic stroke by promoting the phosphorylation of GSK3*β* [15]. Consistent with previous studies, we found a significant upregulation of p-GSK3*β* expression in the brain after ICH, which suggests that endogenous upregulation does not completely reverse brain injury in ICH. The phosphorylation level of GSK3*β* was significantly increased after intranasal administration of HLY78, an agonist of LRP6. Sirt1 is reportedly a major regulator of mitochondrial biogenesis and lipid metabolism and has been shown to be necessary for promoting oxidative stress-induced MEF cell death [47, 48]. It interacts with GSK3*β*, and Koga et al. similarly found that upregulation of Sirt1 can be mediated by GSK3*β* [22, 23]. Sirt1 activation reportedly improves mitochondrial function and reduces brain damage by increasing the level of PGC-1*α* [19, 21]. PGC-1*α* is involved in cellular energy and plays a key role in regulating mitochondrial production and antioxidation [18, 49]. Furthermore, Cordani et al. found that PGC-1*α* could reduce ROS production by regulating UCP2 levels and by protecting mitochondria from ROS-induced damage [50]. To further validate this possible underlying mechanism, we administered LRP6 siRNA and selisistat, a Sirt1 selective antagonist, concomitantly with HLY78 treatment. They reversed the HLY78 on downstream proteins, including GSK3*β*, Sirt1, PGC-1*α*, cleaved caspase-3, and Romo-1. Thus, these observations suggest that the antioxidative and antiapoptotic effects of HLY78 were, at least in part, through the LRP6/GSK3*β*/Sirt1/PGC-1*α* signaling pathway.

The present study has several limitations. First, we applied LPR6 siRNA to knock down LRP6 in the brain in this study, and a transgenic animal model is needed to further validate our findings. Second, we focused only on the GSK3*β*/Sirt1/PGC-1*α* axis as the underlying mechanism of neuroprotection provided by HLY78 after ICH and could not exclude other molecular signaling pathways associated with the benefits of LRP6. Finally, gender and age differences were not assessed in this study.

## 5. Conclusion

In conclusion, the activation of LRP6 with HLY78 improved neurological deficits by attenuating oxidative stress and neuronal apoptosis after ICH. The antioxidative and antiapoptotic effects were, at least in part, through the GSK3*β*/Sirt1/PGC-1*α* signaling pathway. Thus, LRP6 may serve as a novel therapeutic target to ameliorate SBI after ICH.

## Data Availability

The data support the findings of this study and are available from the corresponding author upon reasonable request.
